# *BCOR-CCNB3* sarcoma with concurrent *RNF213-SLC26A11* gene fusion: a rare sarcoma with altered histopathological features after chemotherapy

**DOI:** 10.1186/s12957-022-02611-4

**Published:** 2022-05-14

**Authors:** Wen Huang, Wei Wang, Liang-Liang Huang, Heng Li, Wen-Chao Zhou, Hai-Bo Wu

**Affiliations:** 1 Department of Pathology, the First Affiliated Hospital of USTC, Division of Life Sciences and Medicine, University of Science and Technology of China, Hefei, 230036 Anhui China; 2 Intelligent Pathology Institute, the First Affiliated Hospital of USTC, Division of Life Sciences and Medicine, University of Science and Technology of China, Hefei, 230001 Anhui China

**Keywords:** *BCOR-CCNB3*, Gene fusion, Ewing-like sarcomas, *RNF213-SLC26A11*, Soft tissues

## Abstract

**Background:**

Chemotherapy is a common approach for cancer treatment, but intrinsic genetic mutations in different individuals may cause different responses to chemotherapy, resulting in unique histopathological changes. The genetic mutation along with the distinct histopathological features may indicate new tumor entities. BCOR-CCNB3 sarcomas is a kind of Ewing-like sarcomas (ELS) occurring mostly in bone and soft tissues. No gene fusion other than BCOR-CCNB3 has been found in this type of tumor.

**Case presentation:**

We herein report a case of 17-year-old male patient, presented with a mass on his left shoulder that was diagnosed as undifferentiated small round cell sarcoma according to core biopsy. The patient received 5 courses of preoperational chemotherapy, and the tumor was resected and analyzed. Primitive small round cells and larger myoid cells in the resected tumor tissue but not in biopsy were observed, and arterioles stenosis and occlusion were also detected, indicating a dramatic change of histopathological features of this tumor. In addition, the immunohistochemical results showed the altered staining patterns of BCOR, bcl2, CyclinD1, TLE1, AR, SMA, CD117, STAB2, CD56, and CD99 in tumor tissues after chemotherapy. Notably, RNA sequencing revealed a RNF213-SLC26A11 fusion in the tumor sample.

**Conclusions:**

The BCOR-CCNB3 sarcoma with RNF213-SLC26A11 fusion may indicate a subset of tumors that undergo histopathological changes in response to chemotherapy. More similar cases in the future may help to clarify the clinical meanings of RNF213-SLC26A11 fusion in BCOR-CCNB3 sarcomas and the underlying mechanisms.

## Background

Primitive small blue round cell tumors (SBRCTs) are malignant soft tissue sarcomas common in children and adolescents. Ewing sarcoma (ES) is the prototypical SBRCT characterized by the fusion of *EWSR1* and a member of the *ETS* family of transcription factors. With the rapid development of molecular diagnostics, new entities of SBRCTs have been recognized. Recent reports described a subset of SBRCTs, Ewing-like sarcomas (ELS), which are morphologically similar to ES. ELS contain a broad spectrum of tumors with different gene fusions, and *CIC*-rearranged sarcomas are as the most common ELS account for about two-thirds of ELS [1]. In 2012, Pierron et al. reported *BCOR-CCNB3* sarcomas are a kind of ELS that often occur in bone and soft tissues [2]. Morphologically, *BCOR-CCNB3* sarcomas are highly cellular sarcomas that are composed of varying spindle and ovoid cells, monomorphic nuclei angulated with finely chromatin and indistinct nucleoli, and prominent delicate capillary network. The stroma showed varying amounts of myxoid and collagen [3, 4], and the morphologic spectrum of *BCOR-CCNB3* sarcomas was quite varied. Increased cell density and obvious pleomorphism were observed in the recurrent and metastatic lesions of *BCOR-CCNB3* sarcomas [3–5]. Heterogeneity in morphology appeared in *BCOR-CCNB3* sarcomas after chemotherapy, with possible changes of immunophenotypes such as decrease or loss of expression of CCNB3 and SATB2 [6–8].

The *BCOR-CCNB3* sarcomas may have genetic changes such as HOX family and NTRK3 up-regulation [4], but no gene fusions other than the *BCOR-CCNB3* fusion have been reported so far. The *RNF213-SLC26A11* gene fusion has only been found in chronic myeloid leukemia [9] and glioma [10]. Here, a *BCOR-CCNB3* sarcoma is described with concurrent *RNF213-SLC26A11* gene fusion, showing unique morphologic features and peculiar immunophenotype after chemotherapy.

## Case presentation

### Case description

**Fig. 1:**
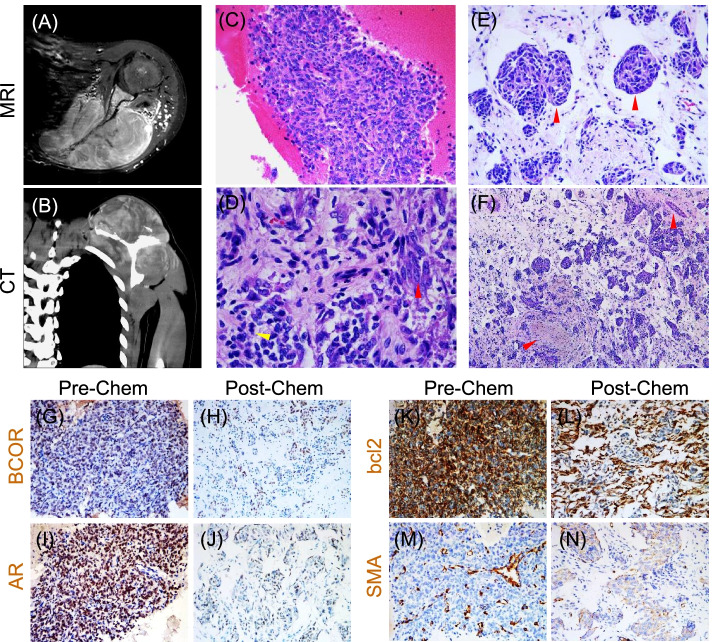
Histomorphological features of the tumor in this study. **A** The MRI imaging showed a huge soft tissue mass growing around the left scapula. **B** The contrast-enhanced CT showed the tumor grew inside and outside the shoulder blade, and the scapular spine. **C** The pre-chemotherapy biopsy showed spindle and ovoid cells that were arranged in solid sheets. **D** The post-chemotherapy tumor showed the primitive small round cells (yellow arrow) and larger myoid like cells (red arrow). **E** Crescent-shaped gaps were noted around the nests, forming a glomerular-like structure in the post-chemotherapy tumor. **F** Vascular stenosis and occlusion were detected in the post-chemotherapy tumor. **G**–**L** IHC staining demonstrated that BCOR, AR, and bcl2 showed diffuse and strong staining in the pre-chemotherapy biopsy (**G**, **I**, **K**), but focal positive mainly in myoid cells in the post-chemotherapy tumor (**H**, **J**, **L**). **M**, **N** IHC showed that SMA expression was negative in most tumor cells in the pre-chemotherapy biopsy (**M**) but positive in myoid cells in the post-chemotherapy tumor (**N**)

A 17-year-old male patient presented a mass on his left shoulder without apparent cause 6 months ago. This patient received no treatment until the mass enlarged with pain and discomfort. MRI showed a huge soft tissue mass (10.0cm×8.1cm×6.6cm) growing around the left scapula with the imaging features of long T1, long T2, and high DWI signal (Fig. 1A). The tumor showed invasive growth and caused irregular left scapula destruction, extending into left supraspinatus, infraspinatus, subscapularis, spina scapulae of deltoid, and other soft tissues. Contrast-enhanced CT showed unevenly mild to moderate enhancement of the mass which grew inside and outside the shoulder blade, and above and below the scapular spine (Fig. 1B). The scapula presented sieve-like bone destruction, while no obvious bone destruction was found in the adjacent ribs, clavicle, and humeral head. Pathological results of core biopsy suggested an undifferentiated small round cell sarcoma without genetic test. The patient received five courses of neoadjuvant chemotherapy (with the regimen of oxaliplatin, pirarubicin, and ifosfamide). After then, he received radical resection of left shoulder blade followed by three circles of ifosfamide chemotherapy. There were no recurrence and metastasis being developed half a year after surgery.

### Immunohistochemical stains

The 3-μm-thick formalin-fixed paraffin-embedded (FFPE) slides were continuously sectioned for immunohistochemical staining of BCOR, Bcl2, CyclinD1, ERG, Fli1, TLE1, AR, Ki67, SMA, Desmin, S-100, STAB2, CD117, CD56, and CD99 with the Roche BenchMark ULTRA fully automated immunohistochemistry stainer.

### Fluorescence in situ hybridization (FISH)

According to the manufacturer’s instruction, FISH was performed on 4-μm-thick FFPE sections (after chemotherapy) with the Dual Color Probe, using Vysis *BCOR* Break Apart FISH Probe kit (Guangzhou LBP Medicine Science & Technology Co., Ltd., China) and Vysis *EWSR1* Break Apart FISH Probe kit (Abbott Molecular, Des Plaines, IL, USA). Two hundred non-overlapping intact nuclei were examined in each slide by a Leica fluorescence microscope. A specimen was considered positive when at least 20% of the nuclei showed a break-apart signal.

### RNA sequencing

Total RNA was extracted from FFPE samples using the RNeasy FFPE Kit (QIAGEN). RNA samples were quantified with BioAnalyzer 2100 (Agilent Technologies), depleted of ribosomal RNA (rRNA) and residual genomic DNA, purified with Agencourt RNA Clean XP Beads, and subjected to the construction of the sequencing library with the KAPA Stranded RNA-Seq Library Preparation Kit. The resultant library was sequenced on Illumina HiSeq X Ten platform (Illumina) for paired-end 150bp sequencing. The outcome in FASTQ format were generated with bcl2fastq v2.16.0.10 software (Illumina), and the somatic fusion genes were identified using FusionCatcher pipeline with default settings (http://code.google.com/p/fusioncatcher/) [11]. The GRCh37/hg19 build was used as the human genome reference.

### Reverse transcription polymerase chain reaction (RT-PCR)

RT-PCR was performed using the primers BCOR-E15-F: 5′-TCACGAACGAAATTCAGACTC-3′ and CCNB3-E5-R: 5′- GCTACTACTGGTGTGACTTCC-3′ for detecting BCOR-CCNB3 fusion, and RNF213-E2-F: 5′-AGGAGGAAACCCCCAAGTTC-3′ and SLC26A11-E8-R: 5′-TCGAAGGAGTACGCAACCAG-3′ for detecting RNF213-SLC26A11 fusion. For Sanger sequencing, BCOR-E15-F and RNF213-E2-F were used.

## Results

### Altered histopathological features of the tumor after neoadjuvant chemotherapy

In the initial core biopsy specimen collected before chemotherapy, the tumor cells were arranged in solid sheets, with the cell morphology of round or oval, eosinophilic cytoplasm, fine chromatin, indistinct nucleoli, and rich capillary network (Fig. 1C). After neoadjuvant chemotherapy, the resected specimen showed infiltration and destruction of bone and muscle. Notably, the morphology and structure of the resected specimen were completely different from those of core biopsy. The tumor cells had a biphasic appearance (Fig. 1D) indicating primitive small round cells (usually distributed in small clusters) with scant cytoplasm, round nucleus, fine chromatin and inconspicuous nucleolus, and larger myoid cells with abundant eosinophilic cytoplasm, oval nucleus, rough chromatin, and obvious small nucleolus. A local transition between the two types of cells was observed. The tumor cells were arrayed in various structures, such as solid, nested, trabecular, and cords-like. Some crescent-shaped gaps appeared around the nests and formed a glomerular-like structure (Fig. 1E). The stroma showed varying degrees of edematous or hyaline degeneration, focal hemorrhage and small focal necrosis of tumor cells, and scattered mast cell infiltration. Besides capillary, arterioles with obvious thickening walls, varying degrees of hyaline degeneration, vascular stenosis, and occlusion were also detected (Fig. 1F).

The immunohistochemical staining showed the diffusely and strongly expression of BCOR, bcl2, CyclinD1, TLE1, and AR in the biopsy specimen, but these markers were focal positive mainly in myoid cells in the radical specimen (Fig. 1G–L). For SMA (Fig. 1M, N) and CD117, the staining was negative in the biopsy but positive in the resected tumor, mainly in myoid cells. STAB2, CD56, and CD99 were scattered positive in few cells in the biopsy tissue, but focal positive in myoid cells in the resected tumor. The proliferation index of Ki67 decreased from 30 to 10% after neoadjuvant chemotherapy. The immunohistochemistry changes in tumor core biopsy and radical specimen are summarized in Table 1.

**Table 1:** The histological and immunohistochemocal changes before and after chemotherapy

IHC	Pre-chemotherapy	Post-chemotherapy	
		Small round cells	Larger myoid cells
BCOR	+	−	+
Bcl2	+	−	+
AR	+	−	+
SMA	−	−	+
SATB2	Scatter +	−	+
CD117	−	−	+
CD56	Scatter +	−	+
CD99	Scatter +	−	+
CyclinD1	+	−	+
TLE1	+	−	+

*IHC* Immunohistochemistry, *BCOR* BCL6 corepressor, *AR* Androgen receptor, *SMA* Smooth muscle actin, *SATB2* special AT-rich sequence-binding protein 2, *TLE1* Transducin-like enhancer protein 1

### FISH analysis of EWSR1 status

**Fig. 2:**
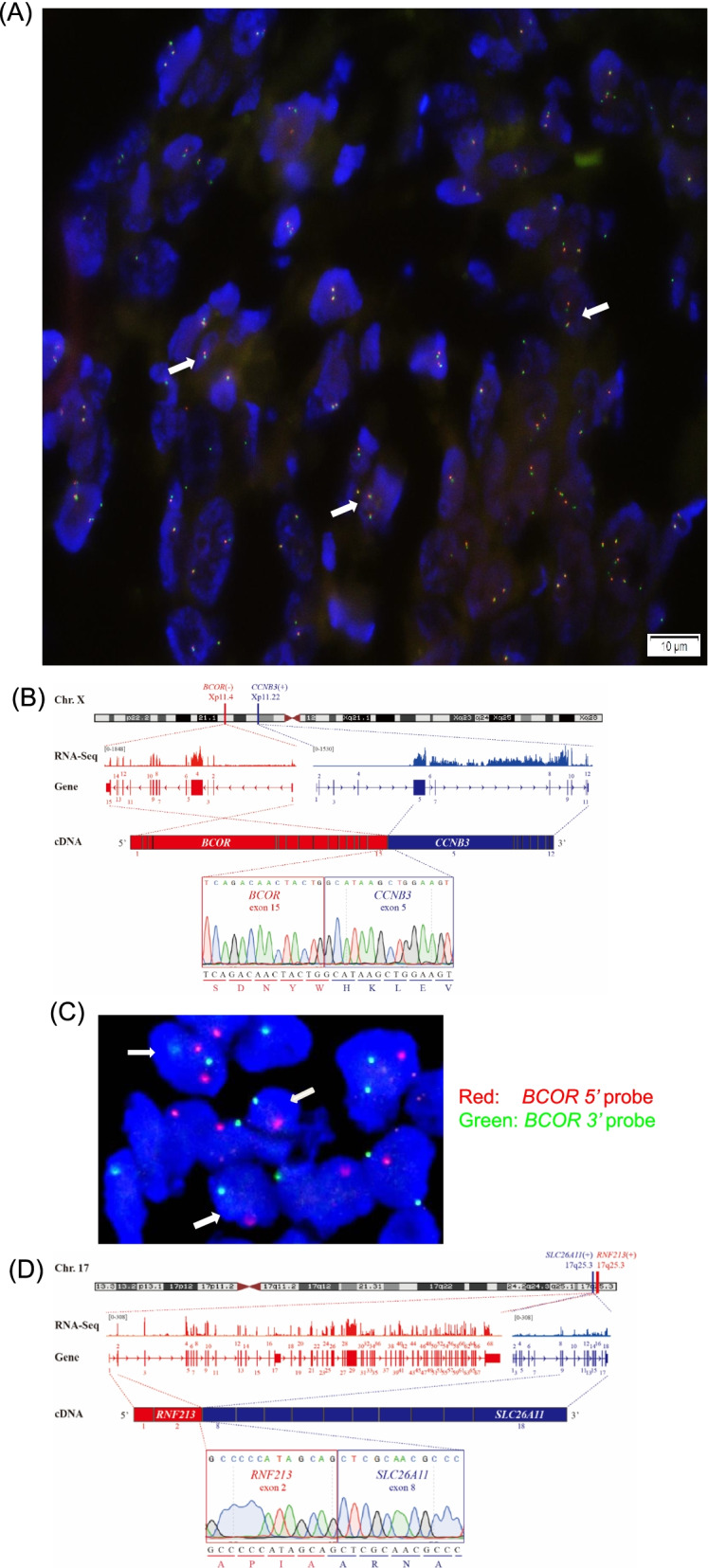
*RNF213-SLC26A11* in addition to *BCOR*-*CCNB3* fusion in the tumor in this study. **A** FISH analysis (with the Vysis EWSR1 (22q12) Dual Color) showed that no split signal of EWSR1 was detected (white arrows). **B** Schematic diagram (RNA sequencing and Sanger sequence of RT-PCR) showed a gene fusion connecting *BCOR* exon 15 to *CCNB3* exon 5 in this tumor. **C** FISH study using *BCOR* break-apart probe demonstrated split of the red (5′) and green (3′) probes (arrows). **D** RNA sequencing and Sanger sequence of RT-PCR revealed a novel *RNF213-SLC26A11* fusion connecting *RNF213* exon 2 to *SLC26A11* exon 8 in this *BCOR*-*CCNB3* sarcoma

According to the FISH analysis (with the Vysis EWSR1 (22q12) Dual Color), a 2-fusion signal pattern could be observed (Fig. 2A) in the specimen after chemotherapy, reflecting the 2 intact copies of EWSR1. This result indicated no split signal of EWSR1 was detected in this patient. For a precious diagnosis, RNA sequencing was performed in the analysis of the mutation status of this patient.

### RNA sequencing revealed the RNF213-SLC26A11 fusion in addition to the BCOR-CCNB3 gene fusion

The result of RNA sequencing showed that no fusion genes related to Ewing’s sarcoma (including EWSR1-FLI1, EWSR1-ERG, EWSR1-ETV1, EWSR1-ETV4, EWSR1-FEV, FUS-ERG, FUS-FEV) were detected. Combined with FISH analysis of EWSR1 (mentioned above), the diagnosis of Ewing’s sarcoma could be excluded.

RNA sequencing identified a *BCOR-CCNB3* gene fusion in this case. This fusion joined the exons 1–15 of *BCOR* (ENST00000342274) to the exons 5–12 of *CCNB3* (ENST00000276014) (Fig. 2B). In the tumor relative to normal control tissues, the reads of the *BCOR* exons showed a sharp decline at the 3′ end (average: ~1000, 3′ end: ~100), and almost no read was observed in the first four exons of *CCNB3*.

To validate the RNA sequencing results, primers covering *BCOR* exon 15 (BCOR-E15-F) and *CCNB3* exon 5 (CCNB3-E5-R) were designed and RT-PCR was performed to obtain the fusion fragment. Sanger sequencing showed that the fusion joined the regular acceptor site of *CCNB3* exon 5 to the putative GGTGAG donor splice-site (just before the stop codon TGA) of *BCOR*, generating a *BCOR-CCNB3* fusion protein. The break-down of the *BCOR* gene at the genomic level was validated by FISH (Fig. 2C). Notably, we discovered a novel *RNF213-SLC26A11* fusion in this case. In detail, the *RNF213* (ENST00000582970) exons 1–2 were joined to the *SLC26A11* (ENST00000411502) exons 8–18, generating an in-frame *RNF213-SLC26A11* isoform (Fig. 2D). RT-PCR and the subsequent Sanger sequencing proved the existence of this novel fusion gene.

## Discussion and conclusions

The *BCOR* gene located at Xp11.4 encoded the ubiquitously expressed BCOR protein (interacts with BCL6) to enhance BCL6-mediated transcriptional repression [2, 4, 12]. Physiologically, BCOR plays an important role in pluripotency by regulating differentiation and cell fate determination [2]. In bone and soft tissue sarcomas, *BCOR* gene mutated mainly through internal tandem duplications (ITD) and gene fusion, among which the *BCOR-CCNB3* is the most common fusion. *CCNB3*, mainly expressed in the germ cells of testis, is a member of the cyclin B family [4]. In addition to *CCNB3*, *BCOR* has been reported to be fused with *ZC3H7B*, *CIITA*, *MAML3*, and *KMT2D* [13]. However, no fusion gene other than *BCOR-CCNB3* has been reported in *BCOR-CCNB3* sarcoma so far. The *BCOR-CCNB3* sarcoma with *RNF213-SLC26A11* gene fusion is the first discovered case.

The *BCOR-CCNB3* sarcoma is more common in men, mainly in older children and adolescents, and it is more in bone than in soft tissue [4–6]. The core biopsy of the *BCOR-CCNB3* sarcoma with *RNF213-SLC26A11* gene fusion is similar to regular *BCOR-CCNB3* sarcomas in terms of histology. Regular *BCOR-CCNB3* sarcoma is often composed of round or oval cells in different proportions, distributing in solid sheets, with less cytoplasm, fine chromatin, indistinct nucleoli, and rich capillary network [4]. Other morphological features in this tumor include myxoid spindle cell component, solid areas of high cellularity alternating with less cellular areas, hemangiopericytoma-like pattern, whorls-like arrangement, trabeculae or cord formation, heterologous cartilaginous differentiation, and fascicular architecture reminiscent of infantile fibrosarcoma [6, 7, 14–17].

*BCOR-CCNB3* sarcomas treated with neoadjuvant chemotherapy are similar to the untreated samples in morphology, but they display hypocellular loose fibrous tissue. Sometimes, there are spindle cells with slit-like spaces and extravasated erythrocytes, foci of extramedullary hematopoiesis, and pleomorphic tumor cells with multi-nucleated cells. Epithelioid cells appear in small clusters or cords, with focally prominent nucleoli [1, 5–8, 18]. However, an undescribed morphological change (*RNF213-SLC26A11* gene fusion) was observed in the *BCOR-CCNB3* sarcoma after neoadjuvant chemotherapy. Most noticeably, two new types of cells, the small round cells similar to classic ES and the larger myoid cells resembling smooth muscle cells or myoepithelioma cells, present in the resected tumor tissue but not the biopsy. The myoid cells showed SMA staining, indicating myogenic differentiation. In addition, we observed a large number of arterioles with thickened or even occluded walls in this tumor, which has never been reported in *BCOR-CCNB3* sarcomas. Our discovery may extend the morphological spectrum of *BCOR-CCNB3* sarcomas after chemotherapy.

So far, there have been no commonly accepted immunohistochemical markers for *BCOR-CCNB3* sarcomas. The diffuse positive expression of CCNB3 can help diagnosis, but the focal or weakly positive staining of CCNB3 occurs in solitary fibrous tumor (SFT), rhabdomyosarcoma, ES, and fibrosarcoma [6]. Although BCOR is expressed in almost all the *BCOR-CCNB3* sarcomas [6], its specificity is doubtful. BCOR also expresses in SFT, synovial sarcoma, ES, malignant lymphoma, and small cell carcinoma [6]. Even with the co-staining of CCNB3 and BCOR, the diagnosis could not exclude the possibility of SFT and ES. Androgen (AR) expression has been detected in various benign and malignant soft tissue tumors, including juvenile nasopharyngeal angiofibroma [19], undifferentiated pleomorphic sarcoma, fibrosarcoma, leiomyosarcoma, rhabdomyosarcoma, myxoid liposarcoma, and angiosarcoma [20]. We reported for the first time that AR is expressed in *BCOR-CCNB3* sarcomas. The expression of AR in sarcoma often indicates potential progression [20], but it may also be a therapeutic target. The immunohistochemical phenotypes of *BCOR-CCNB3* sarcomas often change after neoadjuvant chemotherapy. For example, CCNB3 and SATB2 often have weakened expression or no expression [6–8, 15, 18]. In this case, the expression of BCOR, bcl2, CyclinD1, TLE1, AR, and Ki67 all decreased after chemotherapy. Interestingly, these markers were preserved in myoid cells completely or partially. Meanwhile, SMA, SATB1, and CD117 expression were detected in resected tumor tissues (after chemotherapy) but not in biopsy specimens. The restricted expression of SMA and SATB2 in myoid cells indicated the differentiation of the tumor in response to chemotherapy. However, there is no clue for the origin of the small round cells.

The *RNF213* gene locates on chromosome 17q25.3, coding for a ring finger protein with ubiquitin ligase and ATPase activities. The *RNF213* gene plays an important role in angiogenesis and inflammatory responses of endothelial cells. *RNF213* is a susceptibility gene for moyamoya and it functions to maintain blood flow in the case of hypotension in the brain. It is also a genetic risk factor for pulmonary hypertension and systemic vascular disease [21]. *RNF213* mutations have been found in many cancers and sarcomas [21], while *RNF213* gene translocation occurs in anaplastic large cell lymphoma and inflammatory myofibroblastic tumor [9]. As a member of the SLC26 family, SLC26A11 is mainly involved in anion transportation and acts on homeostasis and intracellular electrolyte balance [9]. The role of SLC26A11 in disease is unclear. *RNF213-SLC26A11* gene fusion has only been reported in chronic myeloid leukemia [9] and glioma [10], which is related to tumor progression. The expression of RNF213-SLC26A11 fusion protein has not been reported previously, and its function in *BCOR-CCNB3* sarcomas is unknown. Because *RNF213* is involved in angiogenesis and vascular-related lesions, the *BCOR-CCNB3* sarcoma with *RNF213-SLC26A11* gene fusion has the thickening of the arterial wall which has not been described for *BCOR-CCNB3* sarcomas after neoadjuvant chemotherapy. It has been speculated that *RNF213-SLC26A11* gene fusion may participate in the vascular change of the tumor.

*BCOR-CCNB3* sarcomas are relatively rare tumors with no standard therapeutic schedule, but neoadjuvant chemotherapy can effectively improve overall survival (OS) and disease-free interval (DFI) of patients [16]. The commonly used ES chemotherapy regimens extend the OS to 75% in 5 years [4, 16]. Some authors believe that the use of clear cell sarcoma of the kidney (CCSK)-based therapy regimens (which emphasize doxorubicin and do not include ifosfamide) can benefit patients while reducing the toxicity of drugs from ES chemotherapy regimens (which include both doxorubicin and ifosfamide) [22]. Although *RNF213-SLC26A11* fusion has been reported in chronic myeloid leukemia and glioma, the efficacy and safety of the corresponding treatment to *RNF213-SLC26A11* were still unknown in *BCOR-CCNB3* sarcomas. For this reason, the therapeutic strategy was not supposed to be changed. In this case, the neoadjuvant chemotherapy with oxaliplatin, pirarubicin, and ifosfamide was also effective, resulting in no recurrence or metastasis of the tumor half a year post surgery. Therefore, accumulating cases with different regimens may help to evaluate the chemotherapy protocol of *BCOR-CCNB3* sarcomas.

## Data Availability

Data could be accessed on request from the authors.

## References

[1] Li WS, Liao IC, Wen MC, Lan HH, Yu SC, Huang HY (2016). BCOR-CCNB3-positive soft tissue sarcoma with round-cell and spindle-cell histology: a series of four cases highlighting the pitfall of mimicking poorly differentiated synovial sarcoma. Histopathology.

[2] Pierron G, Tirode F, Lucchesi C, Reynaud S, Ballet S, Cohen-Gogo S, Perrin V, Coindre JM, Delattre O (2012). A new subtype of bone sarcoma defined by BCOR-CCNB3 gene fusion. Nat Genet.

[3] Miettinen M, Felisiak-Golabek A (2019). Lui?a Contreras A, Glod J, Kaplan RN, Killian JK, Lasota J: New fusion sarcomas: histopathology and clinical significance of selected entities. Hum Pathol.

[4] Kao YC, Owosho AA, Sung YS, Zhang L, Fujisawa Y, Lee JC, Wexler L, Argani P, Swanson D, Dickson BC (2018). BCOR-CCNB3 fusion positive sarcomas: a clinicopathologic and molecular analysis of 36 cases with comp arison to morphologic spectrum and clinical behavior of other round cell sarcomas. Am J Surg Pathol.

[5] Puls F, Niblett A, Marland G, Gaston CL, Douis H, Mangham DC, Sumathi VP, Kindblom LG (2014). BCOR-CCNB3 (Ewing-like) sarcoma: a clinicopathologic analysis of 10 cases, in comparison with convent ional Ewing sarcoma. Am J Surg Pathol.

[6] Matsuyama A, Shiba E, Umekita Y, Nosaka K, Kamio T, Yanai H, Miyasaka C, Watanabe R, Ito I, Tamaki T (2017). Clinicopathologic diversity of undifferentiated sarcoma with BCOR-CCNB3 fusion: analysis of 11 cases with a reappraisal of the utility of immunohistochemistry for BCOR and CCNB3. Am J Surg Pathol.

[7] Ludwig K, Alaggio R, Zin A, Peron M, Guzzardo V, Benini S, Righi A, Gambarotti M (2017). BCOR-CCNB3 undifferentiated sarcoma-does immunohistochemistry help in the identification?. Pediatr Dev Pathol.

[8] Peters TL, Kumar V, Polikepahad S, Lin FY, Sarabia SF, Liang Y, Wang WL, Lazar AJ, Doddapaneni H, Chao H (2015). BCOR-CCNB3 fusions are frequent in undifferentiated sarcomas of male children. Mod Pathol.

[9] Zhou JB, Zhang T, Wang BF, Gao HZ, Xu X (2013). Identification of a novel gene fusion RNF213?SLC26A11 in chronic myeloid leukemia by RNA-Seq. Mol Med Rep.

[10] Bao ZS, Chen HM, Yang MY, Zhang CB, Yu K, Ye WL, Hu BQ, Yan W, Zhang W, Akers J (2014). RNA-seq of 272 gliomas revealed a novel, recurrent PTPRZ1-MET fusion transcript in secondary glioblas tomas. Genome Res.

[11] Nicorici D, Şatalan M, Edgren H, Kangaspeska S, Murumägi A, Kallioniemi O, Virtanen S, Kilkku O. FusionCatcher – a tool for finding somatic fusion genes in paired-end RNA-sequencing data. bioRxiv. 2014:011650.

[12] Astolfi A, Fiore M, Melchionda F, Indio V, Bertuccio SN, Pession A (2019). BCOR involvement in cancer. Epigenomics.

[13] Yoshida A, Arai Y, Hama N, Chikuta H, Bando Y, Nakano S, Kobayashi E, Shibahara J, Fukuhara H, Komiyama M (2020). Expanding the clinicopathologic and molecular spectrum of BCOR-associated sarcomas in adults. Histopathology.

[14] Alfaro-Cervello C, Andrade-Gamarra V, Nieto G, Navarro L, Martín-Vañó S, de la Torre JP G, Bengoa Caamaño M, Garcia Mauriño ML, Noguera R, Navarro S (2017). Congenital undifferentiated sarcoma associated to BCOR-CCNB3 gene fusion. Pathol Res Pract.

[15] Mantilla JG, Ricciotti RW, Chen E, Hoch BL, Liu YJ (2019). Detecting disease-defining gene fusions in unclassified round cell sarcomas using anchored multiplex PCR/targeted RNA next-generation sequencing-Molecular and clinicopathological characterization of 16 cases. Genes Chromosomes Cancer.

[16] Cohen-Gogo S, Cellier C, Coindre JM, Mosseri V, Pierron G, Guillemet C, Italiano A, Brugieres L, Orbach D, Laurence V (2014). Ewing-like sarcomas with BCOR-CCNB3 fusion transcript: a clinical, radiological and pathological retr ospective study from the Soci¨¦t¨¦ Fran?aise des Cancers de L'Enfant. Pediatr Blood Cancer.

[17] Yamada Y, Kuda M, Kohashi K, Yamamoto H, Takemoto J, Ishii T, Iura K, Maekawa A, Bekki H, Ito T (2017). Histological and immunohistochemical characteristics of undifferentiated small round cell sarcomas as sociated with CIC-DUX4 and BCOR-CCNB3 fusion genes. Virchows Arch.

[18] Shibayama T, Okamoto T, Nakashima Y, Kato T, Sakurai T, Minamiguchi S, Kataoka TR, Shibuya S, Yoshizawa A, Toguchida J, Haga H (2015). Screening of BCOR-CCNB3 sarcoma using immunohistochemistry for CCNB3: a clinicopathological report of three pediatric cases. Pathol Int.

[19] Hwang HC, Mills SE, Patterson K, Gown AM (1998). Expression of androgen receptors in nasopharyngeal angiofibroma: an immunohistochemical study of 24 c ases. Mod Pathol.

[20] Fiorelli A, Ricciardi C, Pannone G, Santoro A, Bufo P, Santini M, Serpico R, Rullo R, Pierantoni GM, Di Domenico M (2011). Interplay between steroid receptors and neoplastic progression in sarcoma tumors. J Cell Physiol.

[21] Wang X, Ye M, Wu M, Fang H, Xiao B, Xie L, Zhu X (2020). RNF213 suppresses carcinogenesis in glioblastoma by affecting MAPK/JNK signaling pathway. Clin Transl Oncol.

[22] Argani P, Kao YC, Zhang L, Bacchi C, Matoso A, Alaggio R, Epstein JI, Antonescu CR (2017). Primary renal sarcomas with BCOR-CCNB3 gene fusion: a report of 2 cases showing histologic overlap with clear cell sarcoma of kidney, suggesting further link between BCOR-related sarcomas of the kidney and soft tissues. Am J Surg Pathol.

